# Surgical Treatment Options and the Subtypes of Cesarean Scar Pregnancy Did Not Affect the Probability of a Subsequent Pregnancy: A Prospective Cohort Study

**DOI:** 10.1002/hsr2.72451

**Published:** 2026-04-22

**Authors:** Yunhui Tang, Jing Gao, Qi Chen, Min Zhao

**Affiliations:** ^1^ Department of Family Planning The Obstetrics & Gynaecology Hospital of Fudan University Shanghai China; ^2^ Department of Medical Laboratory The Obstetrics & Gynaecology Hospital of Fudan University Shanghai China; ^3^ Department of Obstetrics, Wuxi Maternity and Child Health Hospital, Wuxi School of Medicine Jiangnan University Wuxi China; ^4^ Department of Obstetrics & Gynaecology The University of Auckland Auckland New Zealand; ^5^ Department of Gynaecology, Wuxi Maternity and Child Health Hospital, Wuxi School of Medicine Jiangnan University Wuxi China

**Keywords:** cesarean scar pregnancy, fertility, optimal treatment, reproductive outcomes, subtypes of CSP

## Abstract

**Background and Aim:**

Cesarean scar pregnancy (CSP) is rare but poses a potentially life‐threatening complication. There is currently insufficient evidence in the literature regarding the impact of CSP treatment on future fertility due to limited clinical studies on a large scale. Here, we investigated the future reproductive outcomes of women with CSP.

**Methods:**

From December 2019 to July 2023, women diagnosed with CSP at the Hospital of Obstetrics and Gynaecology of Fudan University, Shanghai, China, were followed up by telephone calls. Data on future fertility and outcomes were collected.

**Results:**

Among 302 women, excluding three who did not answer the question, 234 (77.5%) did not express their intention to conceive in future, while only 65 (22%) expressed an intention to conceive again. Among these 65 women intending to conceive, 14 (21.5%) achieved pregnancy. In contrast, among women who did not intend to conceive, 31 (15.4%) achieved pregnancy. After excluding women within 12 months of treatment, the analysis showed no statistical difference in successful conception between women who intended to conceive again and those who did not (*p* = 0.0523). Additionally, among the 45 who achieved pregnancy, 26 resulted in live births. In women with successful conception, there was no statistical difference in the association between CSP treatment or CSP subtype and successful conception (*p* = 0.6913 or *p* = 0.5823).

**Conclusion:**

Our study found that 22% of women with CSP intended to conceive, and 29% of our study cohort had a subsequent pregnancy. There was no association between treatment options or CSP subtype and successful conception. Additionally, the incidence of complications in subsequent pregnancies was comparable to that reported in the literature for women without a history of CSP. Our findings suggest that surgical treatments for CSP may not negatively affect future fertility.

## 1 Introduction

Cesarean scar pregnancy (CSP) is a life‐threatening complication of pregnancy, occurring in approximately 1:1800 to 1:2200 of all pregnancies [1], and up to 1:500 in women with a prior cesarean section [2, 3]. Because of the risk of severe complications, early termination is usually recommended in clinical practice. However, in CSP literature, there is still no “gold standard” treatment option, and management remains a clinical challenge [4, 5]. In clinical practice, medical treatment such as methotrexate and several surgical approaches, including transvaginal resection, laparoscopy, uterine artery embolization (UAE) combined with dilatation and curettage (D&C) with or without hysteroscopy, and hysteroscopy alone, are commonly used [6, 7, 8, 9, 10].

Approximately 40%–70% of women with a history of cesarean section develop cesarean scar defects (or “niches”) [11, 12]. These defects are increasingly recognized as both structural and functional sequelae that may interfere with implantation, promote local inflammation, and cause mechanical disruption, leading to adverse outcomes such as miscarriages [13]. Systematic review studies have recently suggested that niches can impair fertility because they can be a physical barrier [14, 15], and niche resection may improve conception rates in women with subfertility [16] and is safe [15]. These findings indirectly support a possible association between CSP and subsequent fertility outcomes.

Despite the increasing incidence of CSP worldwide, data on subsequent pregnancy outcomes after CSP treatment remain limited. This is largely due to the small sample size and the reluctance of women with a prior CSP to pursue another pregnancy. Prior reports indicate varying rates of reproductive desire post‐treatment, with up to 64% of women opting against future pregnancy [17, 18]. As a result, data on outcomes of subsequent pregnancy are limited, even though the low incidence of recurrence of CSP, miscarriage, ectopic pregnancy, preterm birth, placenta accreta, and uterine rupture in a subsequent pregnancy is reported [19].

Fertility outcomes after initial treatment of CSP are also not well characterized in the literature. A systematic review reported a subsequent pregnancy rate of 74% for surgical treatment and 69% for non‐surgical treatment of CSP [20]. However, that study did not address whether different management approaches affect future fertility (reviewed in [19]). Studies with a relatively small sample size reported that 15%–40% of women failed to conceive after 12 months of trying [17, 21]. Our previous study with a medium sample size similarly found that 14% of women with a history of CSP did not conceive without any contraception use [18].

The management strategies of CSP are entirely dependent on individual cases and different hospital protocols, which may influence fertility outcomes in future pregnancies. CSP has traditionally been divided into two subtypes, type 1 and type 2, based on the implantation site of the gestational sac and the thickness of the remaining myometrium [5, 22, 23]. This classification primarily helps to guide the potential treatment options. More recently, a study further proposed three types of CSP, type 1, type 2 and type 3, according to the surgical management strategy [24]. In contrast, the newer Crossover Sign (COS) classification focuses on the relationship between the gestational sac and the previous scar to better predict the risk of placenta accreta spectrum and uterine rupture [25, 26]. Therefore, we conducted this study to evaluate the reproductive outcomes of women with a history of CSP surgically treated in a tertiary hospital.

## 2 Materials and Methods

The Ethics Committee of The Hospital of Obstetrics and Gynaecology, Fudan University, China, approved this prospective study (reference numbers: KYY2019‐76, KYY2020‐185, and KYY2024‐20). The signed consent form was obtained at the time of recruitment. This study was performed in accordance with the Medical Association Declaration of Helsinki.

### 2.1 Study Population

The Hospital of Obstetrics and Gynaecology, Fudan University, China, is located in Shanghai, a city with a population of 25,000,000. Our hospital is one of the largest Obstetrics and Gynaecology specialized hospitals in the country, ranked among the top three nationally. With a capacity of more than 820 beds, we manage more than 100,000 births and more than 1,000,000 gynecological outpatient visits annually.

**Figure 1:**
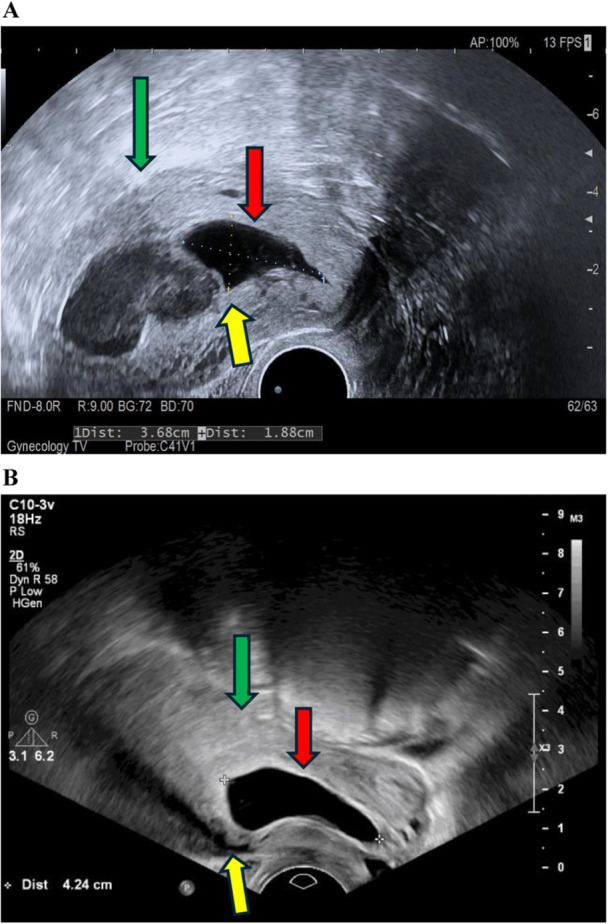
Transvaginal ultrasound images showing type 1 (A) and type 2 CSP (B). The gestational sac (red arrow) is implanted at the site of the previous cesarean scar (yellow arrow), located below and separate from the uterine cavity (green arrow).

This prospective cohort study followed the STROBE guidelines. From December 2019 to July 2023, 345 women were diagnosed with CSP at our hospital and received treatments. CSP confirmation was primarily achieved using transvaginal ultrasound (HITACHI ARTETTA, Japan or Philips) with key diagnostic criteria including the presence of a gestational sac or at least a large part of the gestational sac in the area of the scar, in addition to a history of a prior cesarean section and a positive test of serum β‐hCG. Based on the site of implantation and the gestational sac's growth direction, the CSP was subdivided into two types. Type 1 CSP is when the gestational sac is implanted in the myometrium and growing toward the cervico‐isthmic space or uterine cavity (Figure 1A). Type 2 CSP is when the gestational sac implants deeply into the previous cesarean scar defect and grows toward the myometrium and the uterine serosal layer (Figure 1B) [5, 22, 23]. The initial treatment, mainly performed in our hospital, included ultrasound‐guided suction curettage, ultrasound‐guided suction curettage after uterine artery embolization (referred to as UAE) using a 5‐F angiographic catheter, hysteroscopy or laparoscopy, or a combination of hysteroscopy and laparoscopy. Patients were discharged on Day 2 after treatment and were followed up in outpatient clinics. To confirm successful treatment, postoperative serum hCG testing was performed once a week for 2–4 times. Additionally, a follow‐up ultrasound was performed every 2 weeks for a total of two times.

Clinical data, including age at diagnosis, body mass index (BMI), blood loss during previous treatment, subtype of CSP, initial treatment option, parity, gestational age, the number of surgical abortions, and the interval between the current CSP and the last cesarean section, were collected from our hospital electronic databases.

By the end of November 2023, of these 345 cases, 302 (87.5%) women were followed up by telephone calls. Self‐reported information on a subsequent pregnancy, including pregnancy outcomes, whether intended to conceive again, menstrual cycle length and menses, and menstrual flow, was collected. Not conceiving is defined, according to the World Health Organization (WHO), as the failure to achieve a clinical pregnancy after 12 months or more of regular unprotected sexual intercourse. Therefore, in this study, women who were within 12 months of their initial treatment were excluded from the analysis of the probability of having a subsequent pregnancy.

### 2.2 Statistical Analysis

**Figure 2:**
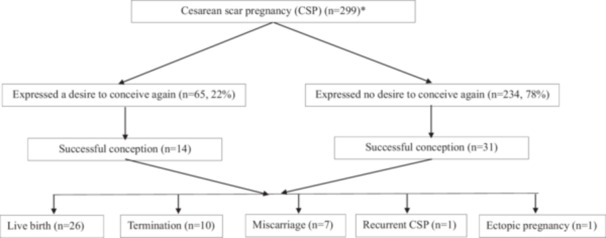
Outcomes of successful conception (*three women did not answer the question regarding the intention of conceiving again).

Among 302 women with CSP who were successfully followed up, 299 provided complete follow‐up data (Figure 2). To assess the efficacy of our sample size, we calculated 95% confidence intervals (CIs) for primary fertility outcomes in subsequent pregnancy. For women who intended to conceive again (*n* = 65), the pregnancy rate was 21.5% (95% CI: 12.5%, 33.3%). While the sample size was determined by the available clinical cohort rather than a prospective power calculation for statistical analysis, the high follow‐up rate (87.5%) and the resulting precision of the CIs allow for a reliable estimate of post‐CSP fertility trends in subsequent pregnancy. Additionally, 43 (12.5%) women were lost to follow‐up. There was no statistical difference in the baseline of clinical parameters, including age at diagnosis, parity, surgical abortions, number of previous cesarean sections and initial CSP subtype between women who were followed up and who lost (*p* > 0.05).

Mean, standard deviation (SD), or percentage were used for data presented in Table 1. To compare the probability of subsequent pregnancy between women who intended and those who did not, or to examine the association between previous treatment options or CSP subtypes and subsequent pregnancy (Tables 2, 3, 4), Chi‐square tests were performed using GraphPad Prism (version 10.1.2). Statistical significance was considered when *p* < 0.05.

**Table 1:** Clinical parameters of the study cohort who were followed up (*n* = 302).

Age at diagnosis of CSP (years, mean/SD)	35.2 ± 4
BMI (Kg/m^2^, mean/SD)	22.73 ± 3.4
Parity (*n*, %)	
1	164 (54%)
2	127 (42%)
≥ 3	11 (4%)
Surgical abortions (*n*, %)	
0	26 (9%)
1	116 (38%)
2	96 (32%)
≥ 3	64 (21%)
Number of previous cesarean sections (*n*, %)	
1	177 (59%)
2	116 (38%)
≥ 3	9 (3)
Subtypes of CSP (*n*, %)	
Type 1	123 (41%)
Type 2	179 (59%)
Treatment options (*n*, %)	
Suction curettage	196 (65%)
UAE	85 (28%)
Others*	21 (7%)
Blood loss during operation (ml, mean/SD)	40 ± 212
Pelvic pain after the previous operation	
No change	56 (18.5%)
No pelvic pain	231 (76.5%)
Had pelvic pain	15 (5%)
Menstrual cycle after the previous operation	
Menses (days, mean/SD)	5 (± 1.7)
Days between cycles	28 (± 3)

* Other referred to as hysteroscopy or laparoscopy or a combination of hysteroscopy and laparoscopy.

**Table 2:** The comparison of subsequent pregnancy in CSP women who intend to have a pregnancy and who did not.

Pregnancy intention after CSP treatment	Became pregnant (*n*, %)	Did not become pregnant (*n*, %)	*p* value (Chi‐square test)
Intended to conceive (*n* = 49)*	14 (28.6%)	35 (71.4%)	*p* = 0.0317
Did not intend to conceive (*n* = 201)*	31 (15.4%)	170 (84.6%)	

* Women within 12 months after initial CSP treatment were excluded.

**Table 3:** The association of previous treatment options and subsequent pregnancy.

Previous treatment options	Became pregnant (*n*, %)	Did not become pregnant (*n*, %)	*p* value (Chi‐square test)
Suction curettage (*n* = 196)	33 (16.8%)	163 (83.2%)	0.6913
UAE (*n* = 85)	7 (8.2%)	78 (91.8%)	
Others (*n* = 21)	5 (23.8%)	16 (76.2%)	

*Note:* Other referred to as hysteroscopy or laparoscopy or a combination of both; UAE: ultrasound‐guided suction curettage after uterine artery embolization (UAE).

**Table 4:** The comparison of subsequent pregnancy outcomes between women with different types of previous CSP.

Overall	Type 1 CSP (***n*** = 123)	Type 2 CSP (***n*** = 179)	*p* value (Chi‐square test)
Became pregnant (*n*, %)	20 (16.3%)	25 (14.0%)	0.5823
Did not become pregnant (*n*, %)	103 (83.7%)	154 (86.0%)	
Live birth (*n* = 26)			
With live birth (*n*, %)	9 (7.3%)	17 (9.5%)	0.5069
Without live birth (*n*, %)	114 (92.7%)	162 (90.5%)	

## 3 Results

Of the 345 women diagnosed with CSP, 302 (87.5%) were successfully followed up. The general clinical characteristics of these women are listed in Table 1. The mean age at diagnosis was 35.2 (± 4) years, and the median age at diagnosis was 35 years, ranging from 24 to 47 years. The mean BMI was 22.73 (± 3.6) Kg/m^2^, and the median BMI was 22.2 Kg/m^2^ (ranging from 16 to 36). The mean blood loss during the previous operation was 40 ml (± 212), and the median was 10 mL, ranging from 1 to 3500 mL. 123 (41%) women were diagnosed with type 1 CSP, and 196 (65%) received suction curettage.

Among the 302 women who were followed up, 3 did not indicate their future reproductive intentions. Of the remaining 299 women, 234 (78%) did not intend to conceive again, while 65 (22%) expressed intention to conceive again (Figure 2). 14 of these 65 women who intended to conceive again achieved pregnancy. After excluding 16 women within 12 months of their initial CSP treatment, the conception rate in the remaining 49 women was 28.6% (Table 2). In contrast, among women who did not intend to conceive again (*n* = 201, excluding 33 women within 12 months of treatment), 31 (15.4%) “accidentally” achieved pregnancy. A Chi‐square test showed a significantly higher proportion of women who intended to conceive and did so than of those who did not intend to conceive but did (28.6% vs. 15.4%, *p* = 0.0317, Table 2).

We next evaluated whether different treatment options for the initial CSP influenced the likelihood of subsequent pregnancy, regardless of whether women intended to conceive again or not. Among the 45 women who achieved pregnancy, 33 (73.3%) had previously undergone suction curettage, 7 (15.6%) underwent UAE followed by suction curettage, and 5 (11.1%) underwent hysteroscopy, laparoscopy, or both. As shown in Table 3, there was no statistical association between prior CSP treatment options and successful conception (*p* = 0.6913). Additionally, we further analyzed whether the subtypes of CSP were associated with successful conception. As shown in Table 4, no significant differences were observed between type 1 and type 2 CSP in achieving either a subsequent pregnancy (*p* = 0.5823) or a live birth (*p* = 0.5069). Detailed clinical parameters, including previous treatment options, parity, history of termination, and the interval between the last CSP and the current pregnancy, for the 45 women who achieved pregnancy are summarized in Table S1.

We also compared the clinical parameters of the previous CSP between women who intended to conceive and achieved pregnancy (*n* = 14) and those who intended to conceive but did not achieve pregnancy (*n* = 35) (Table 5). Although statistical testing was not performed due to the small sample size in some subgroups, no clear differences were observed in previous CSP treatment options, subtype distribution, parity, or number of prior surgical abortions. However, women who did not achieve pregnancy tended to report light menstrual bleeding more frequently than those who conceived (54% vs. 29%).

**Table 5:** Clinical parameters in women who intended to conceive again.

Parameters	Intended & achieved pregnancy (*n* = 14)	Intended but did not achieve pregnancy (*n* = 35)#
**Treatment options for previous CSP**		
Suction curettage	11 (78%)	21 (60%)
UAE	1 (7%)	11 (31%)
Others*	2 (14%)	3 (9%)
**Previous surgical abortions**		
Once	7 (50%)	27 (77%)
Twice	7 (50%)	7 (20%)
≥ Three		1 (3%)
**Subtype of previous CSP**		
Type 1	10 (71%)	14 (40%)
Type 2	4 (29%)	21 (60%)
**Parity**		
One	8 (57%)	22 (63%)
≥Two	6 (43%)	13 (37%)
**Menstrual bleeding pattern after previous CSP treatment**		
Light	4 (29%)	19 (54%)
Same as before	10 (71%)	16 (46%)

* Other referred to as hysteroscopy or laparoscopy, or a combination of hysteroscopy and laparoscopy; UAE: ultrasound‐guided suction curettage after uterine artery embolization.

^#^ Women within 12 months after initial treatment were excluded.

Among the 45 women who achieved pregnancy, 26 had a live birth, 10 requested an elective abortion, 7 experienced early pregnancy loss, 1 developed recurrent CSP, and 1 experienced an ectopic pregnancy (Figure 2). None of the women who had live births developed major obstetric complications such as preeclampsia, gestational diabetes mellitus, or preterm birth.

## 4 Discussion

In this medium‐term follow‐up study (up to 48 months) with a relatively large sample size (*n* = 302), we found that the subsequent pregnancy rate was 28.6% among women who intended to conceive again, excluding those within 12 months of initial CSP treatment. While 15.4% of women who did not intend to conceive again but achieved pregnancy. The successful conception or live birth was not associated with the subtypes of CSP and previous treatment options.

CSP is a rare but potentially life‐threatening complication of pregnancy, with high risks of catastrophic uterine rupture and massive hemorrhage, as well as placenta accreta spectrum [27]. Currently, some evidence reports a relatively lower rate of adverse outcomes in subsequent pregnancy in women with a history of CSP. However, a significant number (20%–80%) of women with a history of CSP do not intend to conceive again, which varies by ethnicity [17, 21, 28]. Our current follow‐up study also showed that 78% of women did not intend to conceive again. Although parity could be one factor influencing the intention of contraception, our data showed no difference in parity between women who intended to conceive again and those who did not (data not shown). Therefore, a higher number of women with a history of CSP did not intend to conceive again, resulting in a lack of evidence about the outcomes of subsequent pregnancy, including subfertility, in the literature.

Increasing evidence recently indicates that the presence of niches caused by cesarean section is potentially associated with infertility. Therefore, whether CSP itself or the treatment options could affect future fertility has not been well answered. Studies reported that 12.5%–40% of women were not pregnant after more than 12 months of attempting to conceive [17, 18, 21, 29]. In our study, in addition to 15.4% of women who did not intend to conceive again but achieved pregnancy, we found a higher proportion (28%) of women who intended to conceive again and did so, after excluding women within 12 months of their initial CSP treatment. Our data may suggest that CSP itself, after treatment, did not influence the probability of a subsequent pregnancy.

Surgical or non‐surgical treatments for CSP are recommended in the current international guidelines. A recent study reported that surgical treatment may cause uterine arteriovenous malformation or uterine artery pseudoaneurysm [30], which may lead to subfertility or recurrent miscarriage [31]. However, a meta‐analysis study reported a 74% subsequent pregnancy rate in CSP after surgical treatments [20]. Our current study found that the successful conception was not associated with the different surgical treatment options, including suction curettage, ultrasound‐guided suction curettage after UAE, hysteroscopy, laparoscopy, or a combination of both (Table 3). Additionally, the number of previous surgical abortions was also not associated with successful conception, even though surgical abortions could damage the endometrium, and the formation of scar tissue in the uterus may impede implantation in the future.

Two types of CSP are traditionally classified based on the site of implantation and the growth direction of the gestational sac [5, 22, 23]. This classification may result in a difference in scar tissue recovery in the uterus after CSP treatment. Whether this difference could affect a subsequent pregnancy has not been investigated. Our current study found that successful conception, including live births, was not associated with CSP subtypes. We also found that the interval between the last CSP treatment and the current pregnancy did not differ between women with different CSP subtypes. Taken together, our data indicate that successful conception was independent of prior surgical treatment and CSP subtypes.

Several factors can influence successful conception. We then compared clinical parameters between women who intended to conceive again and achieved pregnancy and those who did not. Due to the small sample size in some subgroups, our descriptive data showed no clear differences in previous treatment options, parity, history of termination, or interval between the last CSP and the current successful conception between the two groups. However, self‐reported data in the current study showed that a higher number of women with light menstrual bleeding was seen in women who intended to conceive again but did not (Table 5). Women with light menstrual bleeding are associated with a poor conception outcome [32], and changes in the uterine tree could cause dysfunctional menstrual bleeding [33]. A recent follow‐up study (up to 57 months) reported that 60% of women, after an initial treatment with UAE for CSP, experienced reduced menstrual blood volume or amenorrhea [34]. There is currently no evidence indicating CSP treatment results in light menstrual bleeding, suggesting additional research investigating uterine abnormalities and hormone levels in women after CSP treatment would be necessary to definitively establish the impact of CSP and its treatments on future fertility. Additionally, the comparison was not adjusted for other confounders, such as maternal age, inflammation status, and medical history.

Current literature suggests that the incidence of adverse outcomes or complicated pregnancies in subsequent pregnancies is relatively low in women with a history of CSP. Our findings also reported no adverse pregnancy outcomes in those with live births and a lower rate of complicated pregnancies, further suggesting that having a subsequent pregnancy is generally safe in women with a history of CSP.

We acknowledge several limitations in this study. The follow‐up data were obtained from self‐reports, which can introduce response bias [35]. Additionally, the analysis of the association between successful conception and previous CSP treatment options or CSP subtypes has a limitation. The intention to conceive was not controlled when evaluating the association. Some women who did not achieve pregnancy may not have intended to conceive, which could partly influence the observed association. Therefore, the absence of pregnancy in these women may not necessarily indicate reduced fertility potential. Future studies stratified by pregnancy intention could provide a clearer picture of the association. Although our study included a relatively large cohort of 329 women with CSP, the subgroup of women who actively intended to conceive again following CSP treatment was comparatively small (*n* = 65). However, this reflects a well‐documented trend in the literature. Many women after CSP treatment do not intend to conceive again due to the fear of recurrence and the associated psychological impact [18]. In our study, 78% of them chose not to conceive again, which aligns with current evidence.

In conclusion, this medium‐term follow‐up study with a relatively large sample size found that the initial treatment options for CSP or the subtype of CSP were not associated with successful conception in women with a history of CSP. The incidence of complicated subsequent pregnancy was similar to that in women without a history of CSP, as reported in the literature. Our findings suggest that surgical treatments for CSP did not negatively affect future fertility.

## Author Contributions

**Yunhui Tang:** conceptualization, methodology, investigation. **Jing Gao:** investigation, formal analysis. **Qi Chen:** conceptualization, investigation, supervision, writing – original draft, writing – review and editing. **Min Zhao:** conceptualization, writing – review and editing.

## Conflicts of Interest

The authors declare no conflicts of interest.

## Transparency Statement

The lead author Qi Chen, Min Zhao affirms that this manuscript is an honest, accurate, and transparent account of the study being reported; that no important aspects of the study have been omitted; and that any discrepancies from the study as planned (and, if relevant, registered) have been explained.

## Supporting information

Supporting File:

## Data Availability

The datasets used and analyzed during the current study are available from the corresponding author upon reasonable request.
