# Supplementary material for: Surgical Treatment Options and the Subtypes of Cesarean Scar Pregnancy Did Not Affect the Probability of a Subsequent Pregnancy: A Prospective Cohort Study
Source: Health Sci Rep. 2026 Apr 22;9(4):e72451. doi: 10.1002/hsr2.72451 (PMC13103270; doi:10.1002/hsr2.72451)
Supplement: Supplementary file 1 — Supporting File: [file HSR2-9-e72451-s001.doc]

**Supplementary**

**Table 1: Detailed clinical parameters on CSP women with a subsequent pregnancy (n=45)**

| **Pregnancy outcomes** | **Previous subtype of CSP (n, %)** | | **Previous CSP treatment (n, %)** | | **Parity (n, %)** | | **Abortions (n, %)** | | **Interval (years, median/range)** | **Previous cesarean section (n, %)** | |
| --- | --- | --- | --- | --- | --- | --- | --- | --- | --- | --- | --- |
| Live birth (n=26) | Type 1  Type 2 | 9 (35%)  17 (65%) | Suction  UAE  Others* | 18 (70%)  4 (15%)  4 (15%) | One:  Two: | 15 (58%)  11 (42%) | Once  Twice  >twice | 1 (4%)  15 (58%)  10 (28%) | 7 (3-13) | Once  Twice | 15 (58%)  11 (42%) |
| Termination (n=10) | Type 1  Type 2 | 4 (40%)  6 (60%) | Suction  UAE  Others* | 7 (70%)  2 (20%)  1 (10%) | One  Two | 5 (50%)  5 (50%) | None  Once  >twice | 1 (10%)  2 (20%)  8 (80%) | 7 (2-18) | Once  Twice | 5 (50%)  5 (50%) |
| Missed miscarriage (n=7) | Type 1  Type 2 | 6 (86%)  1 (14%) | Suction | 7 (100%) | One  Two: | 3 (42%)  4 (58%) | None  Once  Twice | 1 (14%)  2 (28%)  4 (58%) | 7 (2-13) | Once  Twice | 4 (58%)  3 (42%) |
| Recurrent CSP (n=1) | Type 2 | 1 | Suction | 1 (100%) | Two | 1 | none | 1 | 1 | Twice | 1 |
| Ectopic pregnancy (n=1) | Type 1 | 1 | UAE | 1 (100%) | Two | 1 | Once | 1 | 8 | once | 1 |

*Other referred to as hysteroscopy or laparoscopy or a combination of hysteroscopy and laparoscopy; UAE: ultrasound-guided suction curettage after uterine artery embolization (UAE)
